# Development of a Consensus-Based Cross-Domain Protocol for the Management of Elastic Compression Stocking Therapy in Patients With Deep Venous Thrombosis and Chronic Venous Disease: A Modified Delphi Study

**DOI:** 10.3389/fcvm.2022.891364

**Published:** 2022-05-19

**Authors:** Rachel H. P. Schreurs, Manuela A. Joore, Hugo ten Cate, Arina J. ten Cate-Hoek

**Affiliations:** ^1^Laboratory for Clinical Thrombosis and Haemostasis, Thrombosis Expert Centre Maastricht, Maastricht, Netherlands; ^2^Cardiovascular Research Institute Maastricht (CARIM), Maastricht, Netherlands; ^3^Department of Internal Medicine, Maastricht University Medical Centre, Maastricht, Netherlands; ^4^Department of Clinical Epidemiology and Medical Technology Assessment, Maastricht University Medical Center, Maastricht, Netherlands; ^5^CAPHRI Care and Public Health Research Institute, Maastricht University, Maastricht, Netherlands

**Keywords:** deep venous thrombosis, chronic venous disease, elastic compression therapy, interdisciplinary collaboration, modified Delphi study

## Abstract

**Objective:**

Elastic compression stocking (ECS) therapy is commonly used in patients with deep venous thrombosis (DVT) and chronic venous disease (CVD). The provision of ECS therapy is complex, and studies indicate a lack of practical guidance and suboptimal collaboration among health care professionals. We aimed to reach consensus on critical issues of ECS therapy among the involved health care professionals and patients.

**Methods:**

A three-round modified Delphi analysis was performed in the Netherlands in which 56 health care professionals (internists, dermatologists, general practitioners, emergency room nurses, home care nurses, medical stocking suppliers, and occupational therapists) and seven patients were invited. The 21 statements included in this analysis were based on information collected from a previously conducted Functional Resonance Analysis Method and Realist Evaluation. We used 7-point Likert scale questions and a 75% threshold for consensus.

**Results:**

Of the 63 persons invited for this study, 59 (94%) agreed to participate and responded in the first questionnaire round; of whom 52 were health care professionals and seven were patients (five DVT and two CVD). The overall response rate for the three questionnaire rounds was 91%. After completion of the rounds, full consensus was achieved on 19 out of 21 statements. No consensus was reached on the need for a follow-up appointment for CVD patients and who should be responsible to determine the ECS type (custom-made or standard).

**Conclusion:**

We identified 19 consensus-driven recommendations on treatment decisions and collaboration in ECS therapy among an interdisciplinary panel of health care professionals and patients. These recommendations form a basis for consensus-driven optimization of ECS therapy and should ideally be incorporated in a general cross-domain protocol for ECS therapy in patients with DVT and CVD.

## Introduction

Elastic compression stocking (ECS) therapy is a frequently prescribed treatment worldwide. It is indicated to reduce leg complaints in the acute phase of deep venous thrombosis (DVT) (1–3), to prevent the occurrence of post-thrombotic syndrome which affects up to 50% of DVT patients, and to prevent the development of venous ulcers in patients with chronic venous disease (CVD). Although some controversy remain on the preventive properties of ECS therapy for DVT, the latest Cochrane review indicated a significant reduction in the incidence of the post thrombotic syndrome associated with ECS therapy [RR 0.62 (95% CI = 0.38–1.01, *p* = 0.05)] (4). Elastic compression stocking therapy in CVD has been associated with improved venous symptomatology, oedema, skin changes (5–8), and with the prevention of ulcer recurrence (9). The complications negatively impact patients’ quality of life (10–12). Furthermore, the associated health care burden is substantial and is expected to increase as a result of aging of the global population (especially for CVD) (13–15). Currently, there is no curative treatment for post-thrombotic syndrome once it has occurred, and treatment of venous ulcers is complicated. Prevention of these complications is therefore crucial.

During the process of ECS therapy, patients are confronted with various professionals from different organizations which may affect both the quality and continuity of care. This could hamper patients’ chances to maintain self-reliance during different stages of the process and, subsequently, lead to an increased workload for home care organizations. Two recent studies described the structure of ECS therapy and identified what needs to be in place to maximally support patients and health care professionals to achieve optimal patient outcomes (16, 17). These studies indicated that there was a lack of unambiguous practical guidance regarding management decisions and collaboration among involved health care professionals in local, national, and international guidelines.

To this end, we aimed to reach interdisciplinary consensus on critical management decisions in ECS therapy and to propose consensus-based recommendations for collaboration among health care professionals across various disciplines together with patients involved in ECS therapy.

## Materials and Methods

For this study, we applied a consensus-based approach using a modified Delphi technique. With this technique, sequential questionnaire rounds are presented to an expert panel alternated with controlled feedback on the results collected from the prior rounds. The method allows participants to modify their opinion to achieve consensus regarding a specific topic or process (18, 19). Anonymity between participants is guaranteed, which prevents bias resulting from opinions from dominant participants influencing group opinion (20, 21). We modified the procedure for the qualitative first round as used in the classic Delphi analysis and instead used the content derived from two of our earlier performed studies in this context (a Functional Resonance Analysis method and a Realist evaluation) to develop the questionnaire (16, 17). CREDES criteria were used to conduct and report the study (22).

### Participants

We invited a panel of 56 Dutch health care professionals with a balanced representation of all professional groups involved in ECS therapy (i.e., internists, dermatologists, general practitioners, emergency room nurses, home care nurses, medical stocking suppliers, and occupational therapists) and seven patients (16). The participants included local stakeholders as well as national stakeholders to assure broad support of the consensus reached. National health care professionals were identified based on their involvement in the development of national guidelines regarding the treatment of DVT and CVD, their clinical and/or academic expertise in the field, or if they were representatives of national associations (i.e., the Dutch federation of occupational therapists and the Dutch association for general practitioners) with a special interest in ECS therapy. Experienced patients were purposely selected from the Dutch patient association for patients with cardiovascular diseases, “Harteraad”. Each potential panel member was provided with comprehensive study information, outlining the aim of the modified Delphi analysis, an overview of the elements of ECS therapy, and the extent and timing of their expected involvement as presented in Supplementary Information A.

### Delphi Rounds

We performed a pre-established total of three questionnaire rounds. Each round consisted of statements classified as belonging to the four main stages of ECS therapy as identified by previous research: initial compression, the onset of ECS therapy, implementation of assistive devices, and follow-up (16). For each round, participants were asked to indicate their level of agreement on each of the statements provided on a 7-point Likert scale (1 = strongly disagree, 2 = disagree, 3 = somewhat disagree, 4 = neutral, 5 = somewhat agree, 6 = agree, 7 = strongly agree). All participants were encouraged to provide answers to all questions, a further option “insufficiently informed” was available and participants were asked to use this score if they felt they lacked expertise on a specific topic, this answer option did not influence the level of consensus. In addition, each statement was provided with a textbox to allow participants to provide feedback on statements and/or written justification for their choices. Non-responders were reminded after 1 week. Participants who did not complete a questionnaire by the deadline of the round were considered to have withdrawn from the study and were not invited to take part in subsequent questionnaires. Qualtrics was used to administer, collect, and analyze the online questionnaires.

We used a predefined threshold of 75% of participants indicating at least a score of 6 or higher to reach consensus which is a threshold commonly used for Delphi consensus studies (23). The analysis stopped when consensus was reached for a specific statement, or when the predefined end of study following three rounds was reached. Statements not reaching consensus in round three were deemed non-agreement.

Based on the outcomes of the two studies conducted earlier: the Functional Resonance Analysis Method and the Realist Evaluation, the research team (authors RS, MJ, and AT) developed the draft of the guiding statements for the first round of the modified Delphi study. Statements focused on interdisciplinary collaboration, professionals’ roles and responsibilities, and treatment decisions in ECS therapy. Pilot testing was performed by distributing the draft questionnaire to three independent health care professionals and one patient who were not involved in the study design nor the analysis. Feedback was processed and incorporated in the final draft which comprised demographical questions and 21 statements.

The second round intended to reach further consensus by modification of the statements that lacked consensus as well as to validate possible barriers for implementation based on qualitative information gathered in round 1. These “barrier statements” did not undergo modifications and were only presented to the experts once (round 2 or 3). The third round was used to achieve consensus on the remaining statements and to evaluate possible new barriers that arrived from the qualitative information gathered in round two.

We analyzed both the quantitative level of consensus results (using Microsoft Excel) and qualitative results (content analysis of the text box information) after each questionnaire round. Statements were either dropped or refined, and new statements for subsequent rounds were developed based on the quantitative and qualitative information gathered in the previous round. Participants received anonymized feedback on the results of the previous round at the start of rounds two and three. In this way, we aimed to inform the participants’ judgments and provide them the possibility to amend answers during the next questionnaire round. Additionally, we provided an overview of the statements that already achieved consensus since these statements did not need further consideration in the subsequent round.

## Results

Of the 63 persons invited for this study, 59 (94%) agreed to participate and responded in the first questionnaire round, of whom 52 were health care professionals and 7 were patients (5 DVT and 2 CVD). Fifty-seven participants (97%), including all patients, also responded in the second and third rounds (overall response 91%). Participants’ characteristics can be found in Table 1. Responding health care professionals were equally distributed among the disciplines. Three-quarters of the participants had more than 10 years of clinical experience in the field of either DVT and/or CVD. The majority of health care professionals indicated treating DVT- and/or CVD patients in daily practice, although the number of patients varied.

**TABLE 1:** Background characteristics of the participants responding in the first round.

Healthcare professionals *n* = 52	*N*	%
**Role in the process**		
Health care professional	34	66
A combined health care professional and managerial/policy function	6	12
Managerial/policy function	12	23
**Discipline**		
Vascular internist	6	12
Hematologist	3	6
Dermatologist	7	14
General practitioner	8	15
Medical stocking supplier/skin therapist	8	15
Occupational therapist	6	12
Home care professional	8	15
Emergency room nurse	4	8
Emergency room doctor/resident internal medicine	2	4
**Years’ experience**		
0–5 years	7	13
6–10 years		13
11–20 years	20	38
>20 years	19	36
**Annually treated patients (only for health care professionals or combined functions)**		
*Deep venous thrombosis*		
No patients	2	4
0–25 patients	21	46
25–50 patients	10	22
50–100 patients	8	17
>100 patients	5	11
*Chronic venous disease*		
No patients	5	11
0–25 patients	18	39
25–50 patients	9	20
50–100 patients	8	17
>100 patients	6	13
**Earlier involvement in guideline development**		
Yes	30	57
No	23	43
**Earlier involved as a local stakeholder**		
Yes	10	19
Region Limburg	5	10
Region North-Holland	5	10
No	42	81
**Patients *n* = 7**	***N***	**%**
**Indication elastic compression therapy**		
Deep venous thrombosis	5	71
Chronic venous disease	2	29

**FIGURE 1:**
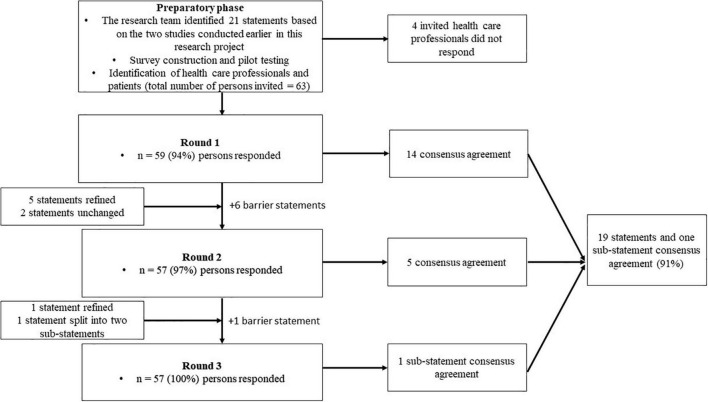
Graphic representation of the modified Delphi process.

**FIGURE 2:**
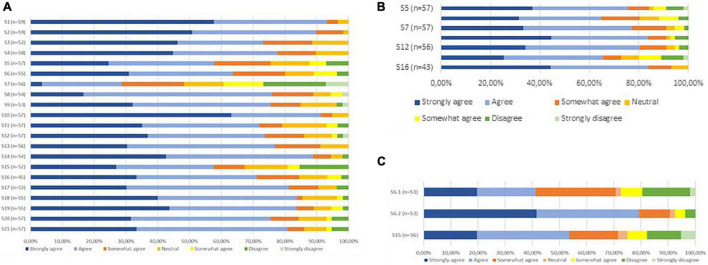
**(A)** Consensus levels statements Round 1. **(B)** Consensus levels statements Round 2. **(C)** Consensus levels statements Round 3.

A graphic representation of the modified Delphi process is presented in Figure 1, and the statements presented to the panelists can be found in Table 2. In the first round, we reached consensus on 14 statements (67%). Of the seven statements for which consensus was not obtained, five were refined and two were resubmitted without modifications in the second questionnaire round. Additionally, we introduced six statements targeting potential barriers for implementation. This questionnaire was sent to the 59 respondents who had responded in the first round. After round two, we reached consensus on five out of seven statements. The two statements for which consensus had not been reached were again refined. We simplified one of these statements (statement 6) by dividing it into two sub-statements which were assessed by the respondents in the last round. Finally, full consensus was reached on 19 statements (91%) and on one of the two sub-statements of statement six. A graphical representation of the consensus levels per round is shown in Figure 2. Detailed information on the statements, adaptations per round, feedback provided to the participants at the start and between each of the questionnaire rounds, and consensus levels can be found in Supplementary Informations B, C.

**TABLE 2:** Final statements and consensus levels per questionnaire round.

Statements
**General statements**
(1) Active involvement of the patient and (if relevant) their informal caregiver in the decision-making process improves the probability of independence in the treatment process
(2) It is important to improve collaboration and dissemination of knowledge among health care professionals involved in elastic compression therapy
**Initial compression therapy**
(3) Both patients with deep venous thrombosis and chronic venous disease (with edema) need to receive initial compression therapy
(4) The treating physician should structurally ask patients about their goals and wishes regarding self-reliance in the process and consider them in the selection of a specific type of initial compression therapy
(5) The treating physician needs to provide general information regarding the options of using assistive devices to maintain self-reliance during the use of elastic compression stockings at the time of diagnosis (either written or oral)
(6) The treating physician is responsible for determining the indication, the pressure class, and the type of ECS. This information should be included in the referral to the medical stocking supplier
(6.1) The treating physician is responsible for determining the indication, the pressure class, and determining if custom made or standard stockings are indicated
(6.2) The treating physician is responsible for determining the indication and pressure class of ECS. This information should be included in the referral to the medical stocking supplier
(7) For patients who do not need home care assistance, the medical stocking supplier needs to assess the presence of edema during the use of initial compression therapy before fitting the ECS*
(8) If home care nurses are involved to apply and remove the initial compression therapy, they are responsible to assess whether the edema has disappeared in direct consultation with the medical stocking supplier. And to subsequently instruct the patient to contact the medical stocking supplier
**The onset of the elastic compression stocking and implementation of assistive devices**
(9) The medical stocking suppliers office should explicitly ask for the presence of edema during the first telephonic contact with the patient
(10) At the moment the elastic compression stocking is delivered, a physical follow-up appointment with the medical stocking supplier needs to take place to fit the stocking and discuss possibilities for self-reliance
(11) The medical stocking supplier is primarily responsible for assessing the patient’s ability to maintain self-reliance in using an assistive device
(12) The medical stocking supplier needs to instruct and train the patient in using an assistive device. If it appears that the patient is not functioning self-reliant at this time, the medical stocking supplier needs to assess whether additional training is useful
(13) It is the medical stocking suppliers (primary) responsibility to discuss the referral to the occupational therapist for additional training with patients who are not directly functioning self-reliant after instruction and training of an assistive device
(14) The most suitable approach to select an assistive device is based on the estimated patient’s physical characteristics and cognitive functioning; goals and wishes; and (if relevant) possibilities to involve the informal caregiver in the process
**Follow-up**
(15) For patients with chronic venous disease, it is important to schedule a follow-up appointment with the treating physician after the elastic compression stocking is delivered (only for the first prescription)*
(16) It is important to individualize deep venous thrombosis patients’ treatment duration with elastic compression stockings based on a risk assessment using Villalta scores with a minimum treatment duration of 6 months
(17) Patients with deep venous thrombosis need to have follow-up appointments with the treating physician until the treatment duration with elastic compression stockings is established (generally after 6 or 12 months)
(18) If the treating physician changes from secondary to primary care during the treatment period, the treating physician should send a letter to the general practitioner which minimally includes the advised treatment duration
(19) All patients should have annual physical follow-up appointments with the medical stocking supplier (as long as the treatment indication lasts) to check and (if necessary) re-measure the elastic compression stocking
(20) If the treating physician changes from secondary to primary care during the treatment period, the first treating physician should inform the patient, general practitioner, and home care organization (if involved)
(21) When elastic compression therapy needs to be discontinued, the treating physician should inform the patient, the medical stocking supplier, and the home care organization (if involved)

*These statements underwent major modifications throughout the questionnaire rounds as presented in Supplementary Information B.

The Delphi panel agreed that collaboration and dissemination of knowledge among health care professionals involved in ECS therapy should be improved (90%) and active patient involvement in the decision-making process is important (93%). Consensus was reached for different treatment decisions concluding that: (1) Initial compression therapy should be offered to all patients (76%); (2) Involvement of the occupational therapist should be accessible to all patients who are not able to maintain self-reliance using an assistive device after instruction and demonstration by the medical stocking supplier (77%); (3) A tailored treatment duration should be implemented for all DVT-patients (84%).

The statement that did not reach consensus concerned the need for a follow-up appointment for CVD patients (statement 15). Although most respondents agreed that a follow-up appointment was necessary, heterogeneity existed in the opinions on who needs to perform these follow-up appointments: the medical stocking supplier or the treating physician. Furthermore, most general practitioners stated that a follow-up appointment was not necessary by default. In their opinion, default follow-up appointments do not add value since in most cases no problems exist. These GPs relied on the patient’s responsibility to contact them if problems occurred.

Only partial consensus was reached for statement 6. Consensus (79%) was reached on responsibility of the treating physician for the determination of the ECS indication and pressure class (sub-statement 6.2). Hence, there was a large heterogeneity among respondents on who should determine the ECS type (sub-statement 6.1) and for this sub-statement consensus was not reached. Some respondents (mainly medical stocking suppliers and general practitioners) indicated that most treating physicians lack the knowledge to determine the correct ECS type. In their opinion, this role should be delegated to the medical stocking supplier. Other respondents stated that treating physicians should determine the type of ECS since financial barriers can influence medical stocking suppliers in selecting the type.

Finally, a high rate of agreement was found for most barrier statements, as represented in Table 3. These barriers included: the treating physicians’ lack of knowledge, reimbursement constraints for assistive devices and training time, administrative burdens, and a lack of expertise regarding applying multilayer compression bandages.

**TABLE 3:** Barrier statements.

	*n*	Strongly disagree *n* (%)	Disagree *n* (%)	Somewhat disagree *n* (%)	Neutral *n* (%)	Somewhat agree *n* (%)	Agree *n* (%)	Strongly agree *n* (%)	Insufficiently informed *n*
(1) There is a lack of knowledge among treating physicians regarding different types of initial compression therapy	46	0 (0)	1 (2)	1 (2)	3 (7)	7 (15)	24 (52)	10 (22)	11
(2) There is a lack of knowledge among treating physicians to inform patients about assistive devices at the moment of diagnosis	52	1 (2)	4 (8)	6 (12)	3 (6)	4 (8)	25 (48)	9 (17)	5
(3) There is a lack of knowledge among treating physicians to determine the appropriate strength and type (circular or flat-knit) elastic compression stocking	46	0 (0)	4 (9)	0 (0)	2 (4)	6 (13)	26 (57)	8 (17)	11
(4) Variable reimbursement criteria are a barrier to optimally select initial compression therapy and assistive devices	43	0 (0)	0 (0)	1 (2)	2 (5)	8 (19)	21 (49)	11 (26)	14
(5) The administrative burden of achieving reimbursement for assistive devices is a barrier to optimal selection	45	0 (0)	2 (4)	0 (0)	5 (11)	9 (20)	16 (36)	13 (29)	12
(6) Home care nurses and staff applying multilayer compression bandages at the general practice lack expertise to apply them with appropriate quality which extends the duration of initial compression therapy	36	0 (0)	4 (11)	5 (14)	3 (8)	10 (28)	9 (25)	5 (14)	21
(7) The need to obtain prior permission from some insurance companies for the implementation of advanced assistive devices to receive coverage and delivery times are a barrier for optimal implementation of these devices	50	1 (2)	1 (2)	0 (0)	2 (4)	2 (4)	19 (38)	25 (50)	7

## Discussion

This study outlines a three-round modified Delphi procedure among experts across different disciplines as well as patients with experience of ECS therapy. We reached full consensus on 19 out of 21 statements (consensus rate 91%). These statements provide recommendations for tangible transitional care agreements that can be used to inform new guidance documents to support treatment decisions and collaboration among professionals involved in ECS care. These recommendations mainly target interdisciplinary collaboration, improving the use of initial compression therapy for all patients, accessible involvement of the occupational therapist at the start of ECS therapy to enhance the patient’s self-reliance, and a tailored ECS treatment duration for DVT patients.

In general, this study shows the clear need for optimization of interdisciplinary collaboration, including dissemination of knowledge, and empowering patients to be involved in their treatment process. The need for this “integrated care” is in line with developments in care worldwide as a response to the fragmented delivery of health care services in current practice (24–26). The World Health Organization states that integrated care is important to ensure interdisciplinary collaboration, optimal outcomes, and appropriate research use (27, 28).

Our Delphi panel agreed that initial compression therapy should be offered to all DVT- and CVD patients. This recommendation is supported by several studies showing the positive short-term effects of immediate initial compression on the occurrence of the post-thrombotic syndrome, reduction of vein occlusion, and reduction of pain and edema for DVT patients (2, 3, 29). However, evidence on the long-term effectiveness was lacking until a sub-study of the IDEAL-DVT showed an 8% absolute reduction for developing the post-thrombotic syndrome after immediate compression (<24 h) at 24 months (30). We could not identify studies specifically evaluating the effects of initial compression therapy for CVD patients, although there is some evidence showing a reduction in symptoms and an improved quality of life associated with compression therapy in general (31, 32).

Furthermore, the panel agreed that the occupational therapist should be actively involved if the patient is not self-reliant after a short introduction and demonstration by the medical stocking supplier. Different experts stated that they expected this intervention to improve the number of self-reliant patients; however, although these expert’ statements are clinically comprehensible, there is a lack of evidence supporting this assumption. Therefore, this could be an important target for future studies.

Finally, our panel agreed that an individualized treatment duration based clinical assessments of the leg is indicated for DVT patients. This is in line with the latest large RCT in the field, showing that this individualized treatment approach is non-inferior compared to standard treatment (33). In the IDEAL-DVT trial, 66% of patients could terminate ECS treatment within 12 months which, in turn, enhanced patients’ self-reliance (especially for patients needing home care assistance).

Reaching consensus on ECS treatment recommendations is the first step toward implementation in daily practice leading ultimately to improved processes and outcomes. Next, a consensus-based protocol endorsed by professional associations and health care authorities should be developed to facilitate implementation into daily practice. Special attention should be focused on health care professionals’ knowledge gaps, reimbursement constraints, and reducing the administrative pressure since panelists rated these topics as important.

Some limitations to our study should be mentioned. Dermatologists accounted for four out of six non-responders. Although this might potentially have led to selection bias, the lack of response did not result in an underrepresentation of this professional group in the analysis. Furthermore, the final draft of the recommendations included in this modified Delphi study was not reviewed and approved by an external board as is recommended by the CREDES criteria (22). We acknowledge that though some of the recommendations in our manuscript are likely to be transferable to other countries, the extent to which they are transferable completely depends on how compression therapy is organized. The major strength of this study is the broad representation of professionals from each participating discipline and contribution of patients, with a balanced composition of the groups, involving both participants with a policy-based background as well as participants with extensive clinical experience. We achieved high overall response rates (91%) as well as high consensus rates (95%) which implies a broad consensus and support base on the content and structure of a general cross-domain protocol to be created. Finally, the panelists had a good understanding of the topic as well as the realities of clinical practice and were therefore able to select elements that should be included in a general cross-domain protocol to optimally support and complement their practice.

## Conclusion

This study identified 19 consensus-driven recommendations for the optimization of ECS therapy in both DVT- and CVD-patients with broad interdisciplinary support. The main topics being: interdisciplinary collaboration, the use of initial compression therapy for all patients, improving patients’ self-reliance, and tailored ECS treatment duration for DVT patients. These recommendations should be included in a general cross-domain protocol as the first step toward implementation in daily practice and are ultimately expected to improve processes and outcomes. Future research should focus on testing the feasibility of the recommendations and cost consequences in daily practice.

## Data Availability Statement

The original contributions presented in the study are included in the article/Supplementary Material, further inquiries can be directed to the corresponding author.

## Ethics Statement

The studies involving human participants were reviewed and approved by the Ethics Board of Maastricht University Medical Center (MUMC), Maastricht (2019-1125), and was considered not to be subject to the Medical Research involving Human Subjects Act (WMO). The patients/participants provided their written informed consent to participate in this study.

## Author Contributions

RS, MJ, and AC-H: study design, development of the guiding statements for Round 1, and data analysis and interpretation. RS, MJ, AC-H, and HC: manuscript writing and revision, read, and approved for submission to Frontiers in cardiovascular medicine.

## Conflict of Interest

The authors declare that the research was conducted in the absence of any commercial or financial relationships that could be construed as a potential conflict of interest.

## Publisher’s Note

All claims expressed in this article are solely those of the authors and do not necessarily represent those of their affiliated organizations, or those of the publisher, the editors and the reviewers. Any product that may be evaluated in this article, or claim that may be made by its manufacturer, is not guaranteed or endorsed by the publisher.
