# Supplementary material for: Development of a Consensus-Based Cross-Domain Protocol for the Management of Elastic Compression Stocking Therapy in Patients With Deep Venous Thrombosis and Chronic Venous Disease: A Modified Delphi Study
Source: Front Cardiovasc Med. 2022 May 19;9:891364. doi: 10.3389/fcvm.2022.891364 (PMC9160232; doi:10.3389/fcvm.2022.891364)
Supplement: Supplementary file 1 [file Data_Sheet_1.pdf]

Supplementary information A: invitation letter and participants information

Dear panelists,

Thank you for participating in our modified Delphi study!

The study is part of an implementation study on elastic compression stocking therapy for patients with deep venous thrombosis, and chronic venous diseases with a long-term indication for elastic compression stockings. A large variety of health care professionals are involved in elastic compression stocking therapy, requiring sufficient coordination and collaboration to achieve optimal outcomes. This study aims to achieve consensus regarding a national cross-domain protocol concerning elastic compression stocking therapy, matching daily practice.

The statements in the survey are based on an earlier performed analysis regarding elastic compression stocking therapy in daily practice in two regions in the Netherlands (Limburg and North-Holland). We kindly ask you to provide your answer based on your profession and work experience. The answers given by you and the other experts will be anonymously used as a basis for the subsequent questionnaires. A total of 3 questionnaire rounds will be performed. The first round aims to rate statements to create a basis for the cross-domain protocol. The second round aims to further deepen understanding of the statements that lacked consensus in the first round and to identify possible barriers for implementation. The last round will be used to achieve consensus on the remaining statements. Using this method, we will be working to a consensus for a national cross-domain protocol for elastic compression stocking therapy. For validity and reproducibility of the results, it is important that you participate in all rounds. The survey will take approximately 15 minutes of your time.

Instructions:

1. We kindly ask you to look at the 'overview of elastic compression therapy' figure added in the supplementary information. We recommend you use the figure to guide you through the time consecutive elements of the survey. Additionally, an explanatory list of medical terms can be found in the supplementary information.
2. Please answer all statements, you will be allowed to add comments after each statement. We encourage you to do so, especially if you do not agree with the statement.
3. If you feel like you lack the information to assess the statement, or it is not within your expertise please choose 'insufficiently informed'
4. The maximum response time for this survey is two weeks.

Explanatory word list:
Compression therapy: the entire process from diagnosis till the end of elastic compression stocking therapy
Edema: the leg contains fluid
Chronic venous disease (stadium C4/C5): the leg contains varicose veins, fluid, and skin changes without an active wound
Treating physician: responsible physician (this could be either the general practitioner, the dermatologist, the physician assistant, specialized nurse or the internist depending on treatment setting)
Initial compression therapy: the initial form of compression therapy aiming to resolve edema (e.g. bandages, temporary compression hosieries, or adjustable compression devices). This type of compression is usually used until the elastic compression stocking is delivered.
Villalta scores: a clinical decision rule (CDR) combining items on patient's complaints, and findings of physical examination of the leg. This CDR makes the diagnosis when for post-thrombotic syndrome (a complication of deep venous thrombosis) when the total score is 5 or higher at least 6 months after the onset of DVT.
Medical stocking supplier/skin therapist: the medical stocking supplier and the skin therapist have the same function in the process.

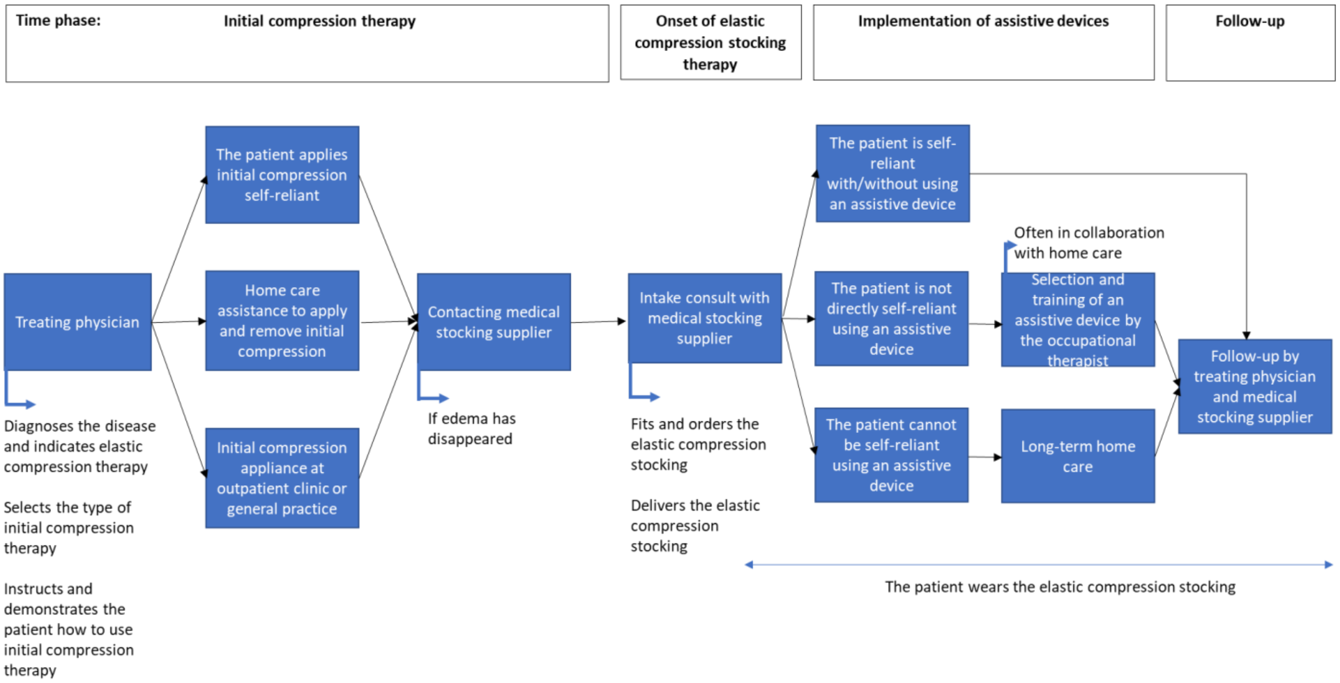

Supplement figure 1. Process of elastic compression therapy: brief overview

Supplementary information B: modified Delphi questionnaires round 1-3

Round 1

<u>Participant Information</u>

Before starting the actual questionnaire we would like to ask you to answer the following questions regarding your background.

Do you agree to participate in this modified Delphi analysis?

- Yes
- No → directed to the end of the survey

Which function do you have in the process of elastic compression therapy?

- Health care professional
- A managerial or policy-related function → directed to how many years of experience in the field of elastic compression stocking therapy do you have?
- Health care professional combined with a managerial or policy-related function
- Patient → directed to for what indication do you / did you wear elastic compression stockings?

How many deep venous thrombosis patients do you assess annually?

- No patients
- 0-25 patients
- 25-50 patients
- 50-100 patients
- > 100 patients

How many chronic venous disease patients (CEAP stadium C4 or C5) who are conservatively treated do you assess annually?

- No patients
- 0-25 patients
- 25-50 patients
- 50-100 patients
- > 100 patients

How many years of experience in the field of elastic compression stocking therapy do you have?

- 0-5 years
- 6-10 years
- 11-20 years
- > 20 years

What is your discipline?

- Vascular medicine
- Hematology

- Dermatology
- General practitioner
- Medical stocking supplier/skin therapist
- Occupational therapy
- Home care
- Emergency room nurse
- Resident internal medicine/emergency room physician

Have you ever been involved in the development of a guideline?

- Yes
- No

For what indication do you / did you wear elastic compression stockings? (Only for patients)

- Deep venous thrombosis
- Chronic venous disease
- I do not know

Have you been involved in the earlier stages of this study as a local stakeholder?

- Yes → directed to the end of participant information
- No

In which region do you work?

- Limburg
- North-Holland

General statements*

The following two statements will be directed at the entire process of elastic compression stocking therapy. If a statement does not include a specific indication (deep venous thrombosis or chronic venous disease), the statement includes both diseases.

1. Active involvement of the patient and (if relevant) their informal caregiver in the decision-making process improves the chance of independence in the treatment process
2. It is important to improve collaboration and dissemination of knowledge among health care professionals involved in elastic compression therapy

Initial compression therapy*

The following statements will be directed at the initial compression phase. From the moment elastic compression therapy is indicated until the moment the patient visits the medical stocking supplier for an intake consult. The timeline indicates the time phase the statement is directed at.

Initial compression therapy	Onset of elastic compression stocking therapy	Implementation of assistive devices	Follow-up

3. It is important that both patients with deep venous thrombosis and chronic venous disease (with edema) receive initial compression therapy
4. It is important that the treating physician structurally asks patients about their goals and wishes regarding self-reliance in the process, and considers them in the selection of a specific type of initial compression therapy
5. The treating physician needs to provide general information regarding the options of using assistive devices to maintain self-reliance during the use of elastic compression stockings at the time of diagnosis
6. The treating physician is responsible for determining the indication, the pressure class, and the type of elastic compression stocking. This information should be included in the referral to the medical stocking supplier

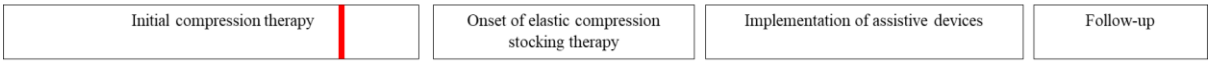

7. Patients who do not require home care assistance for initial compression therapy can assess whether edema has disappeared without the interference of a health care professional, and then make an appointment with the medical stocking supplier
8. If home care nurses are involved to apply and remove the initial compression therapy, they are responsible to assess whether the edema has disappeared and to instruct the patient to contact the medical stocking supplier

<u>Onset of the elastic compression stocking and implementation of assistive devices*</u>

The following statements will be directed at the onset of the elastic compression stocking and the implementation of assistive devices. From the moment the first contact with the medical stocking supplier takes place until the implementation of assistive devices (including training if necessary).

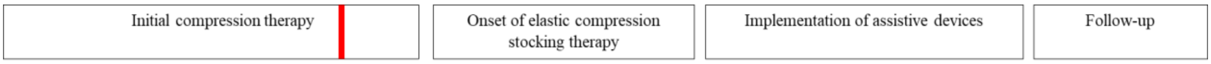

9. It is important that the medical stocking supplier explicitly asks for the presence of edema during the first telephonic contact with the patient

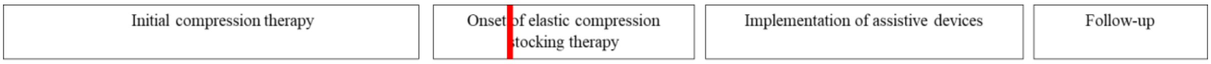

10. At the moment the elastic compression stocking is delivered, a physical follow-up appointment with the medical stocking supplier needs to take place to fit the stocking and discuss possibilities for self-reliance

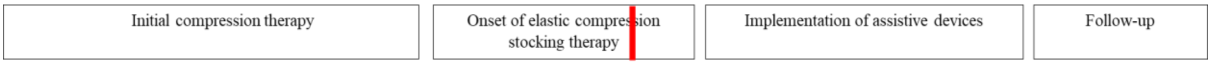

11. The medical stocking supplier is primarily responsible for assessing the patient's ability to maintain self-reliance in using an assistive device

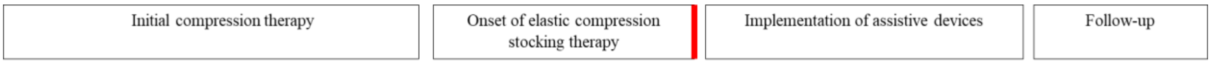

12. The medical stocking supplier needs to instruct and train the patient in using an assistive device. If it appears that the patient is not functioning self-reliant at this time, the medical stocking supplier needs to assess whether additional training is useful

13. It is the medical stocking suppliers (primary) responsibility to discuss the referral to the occupational therapist for additional training with patients who are not directly functioning self-reliant after instruction and training of an assistive device

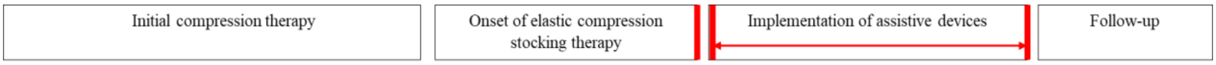

14. The most suitable approach to select an assistive device (with the medical stocking supplier or occupational therapist) is based on the patient’s physical characteristics; goals and wishes, and (if relevant) possibilities to involve the informal caregiver in the process

Follow-up*

The following statements will be directed at the follow-up phase. From the moment the patient applies the elastic compression stocking self-reliantly or with home care assistance until the moment the indication for elastic compression therapy ends (for patients with deep venous thrombosis).

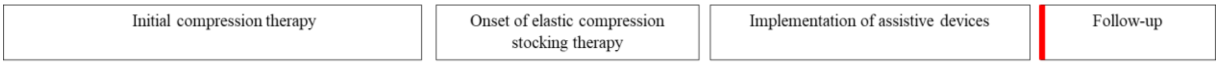

15. For patients with chronic venous disease, it is important to schedule a follow-up appointment with the treating physician after the elastic compression stocking is delivered

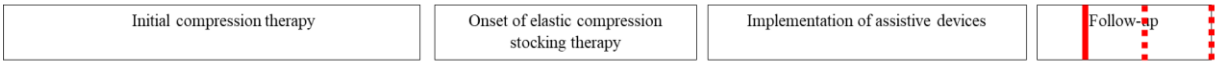

16. It is important to individualize deep venous thrombosis patients’ treatment duration with elastic compression stockings based on a risk assessment using Villalta scores with a minimum treatment duration of six months
17. Patients with deep venous thrombosis need to have follow-up appointments with the treating physician until the treatment duration with elastic compression stockings is established (generally after 6 or 12 months)

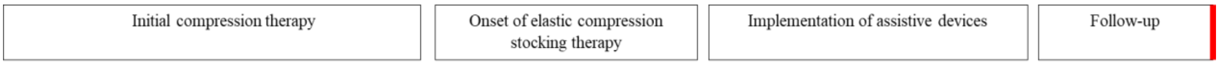

18. When the treatment duration is established, the treating physician should send a letter to the general practitioner which minimally includes any additional follow-up appointments required and the advised treatment duration
19. All patients should have annual physical follow-up appointments with the medical stocking supplier (as long as the treatment indication lasts) to check and (if necessary) re-measure the elastic compression stocking
20. If the treating physician changes during the treatment period, the first treating physician should inform all involved professionals about this change
21. When elastic compression therapy needs to be discontinued (e.g. because of the development of contra-indications or end of treatment duration) the treating physician should inform all involved health care professionals

Thank you for filling out our survey. We will process all answers and soon invite you for the second round of this analysis.

* All statements were provided with the following answer options and an open field text box.

Strongly disagree	Disagree	Somewhat disagree	Undecided	Somewhat agree	Agree	Strongly agree	Insufficiently informed
O	O	O	O	O	O	O	O

Round 2

General information

Dear participant,

We would like to extend our gratitude to everyone for participating in the first questionnaire of our modified Delphi study. We reached consensus on 14 out of 21 statements. These statements are provided in the supplementary file.

In this second questionnaire, you will receive feedback from the non-consensus statements of the first questionnaire. Some statements underwent modifications based on the qualitative responses provided. We kindly ask you to re-assess the statements. Additionally, six statements regarding barriers to implementation were added to the current questionnaire. This questionnaire will take approximately 15 minutes of your time.

Instructions:

5. We kindly ask you to take a look at the ‘overview of elastic compression therapy’ figure added in the supplementary information. We recommend you use the figure to guide you through the time consecutive elements of the survey. Additionally, an explanatory list of medical terms can be found in the supplementary information.
6. Please answer all statements, you will be allowed to add comments after each statement. We encourage you to do so, especially if you do not agree with the statement.
7. If you feel like you lack the information to assess the statement, or it is not within your expertise please choose ‘insufficiently informed’
8. The maximum response time for this survey is two weeks.

Non-consensus statements round 1

The next 7 statements did not receive consensus in questionnaire 1 (defined as < 75% of participants scoring a 6 (agree) or 7 (totally agree) on the Likert score)). Both quantitative and qualitative feedback is given per statement. We ask you to re-assess the statement after reading this feedback.

Initial compression therapy*

Statement 5: It is important that the treating physician provides general information regarding the options of using assistive devices to maintain self-reliance during the use of elastic compression stockings at the time of diagnosis

Respons (n=57)

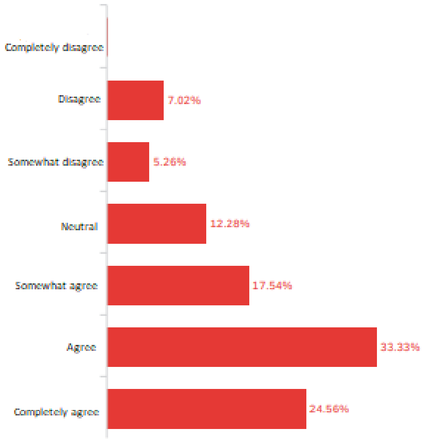

Summary of qualitative responses (n=30):

- 57.9% of the respondents agreed or completely agreed with the statement, including all patients.
- Respondents stated that giving this information early in the process could benefit the patient's acceptance of the ECS and repeating information during the process is necessary.
- Some respondents (n=3, mainly patients) stated that there is a lack of information regarding assistive devices during the entire process.
- Some respondents (n=5) stated that there is a lack of expertise and time to provide this information (especially at the emergency room) and it would be better to assign this task to the medical stocking supplier (n=6).
- Two professionals stated that they were concerned that patients receive too much information at the moment of diagnosis.

Statement 5 (minor adaptation): The treating physician needs to provide general information regarding the options of using assistive devices to maintain self-reliance during the use of elastic compression stockings at the time of diagnosis (either written or oral)

Statement 6: The treating physician is responsible for determining the indication, the pressure class, and the type of elastic compression stocking. This information should be included in the referral to the medical stocking supplier

Respons (n=55)

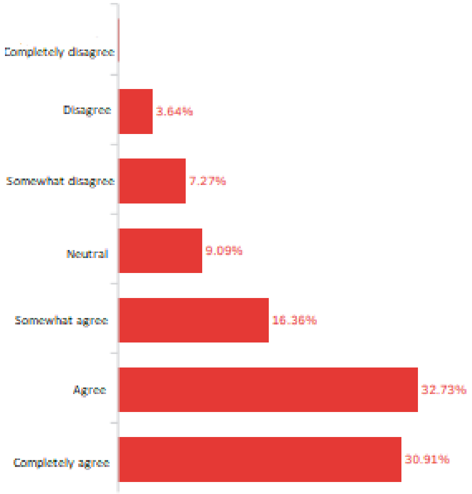

Summary of qualitative responses (n=23)

- 63.6% of the respondents agreed or completely agreed with the statement, of whom 87.5% of the medical stocking suppliers.
- Other respondents from different disciplines (n=12) stated that treating physicians lack the knowledge to determine the appropriate compression strength and type and it would be better to assign this task with the medical stocking supplier.
- One medical stocking supplier indicated that external financial incentives trigger a reticent attitude towards implementing more expensive ECS for medical stocking suppliers.

Statement 6 (without adaptations): The treating physician is responsible for determining the indication, the pressure class, and the type of ECS. This information should be included in the referral to the medical stocking supplier

Statement 7: Patients who do not require home care assistance for initial compression therapy can assess whether edema has disappeared without the interference of a health care professional, and then make an appointment with the medical stocking supplier

Respons (n=56)

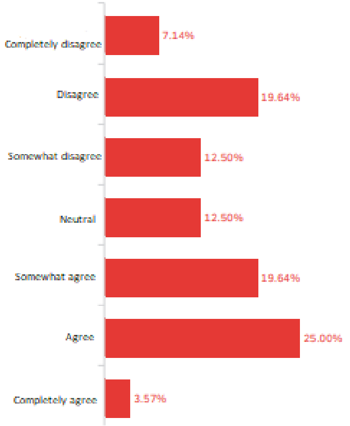

Summary of qualitative responses (n=27):

- The results on this statement vary, resulting in a small consensus rate (28.6%). Only 14.3% of patients and 0% of medical stocking suppliers agreed with the statement.
- Over 50% of respondents from different disciplines indicated that only some patients can assess the edema self-reliant (patients with sufficient cognitive functioning and awareness.
- Approximately 33% of respondents from different disciplines suggested that the medical stocking supplier or treating physician should be responsible to assess the edema.

Statement 7 (major adaptation): For patients who do not need home care assistance, the medical stocking supplier needs to assess the presence of edema during the use of initial compression therapy before fitting the ECS.

<u>The onset of the elastic compression stocking and implementation of assistive devices*</u>

Statement 11: The medical stocking supplier is primarily responsible for assessing the patient's ability to maintain self-reliance in using an assistive device

Respons (n=57):

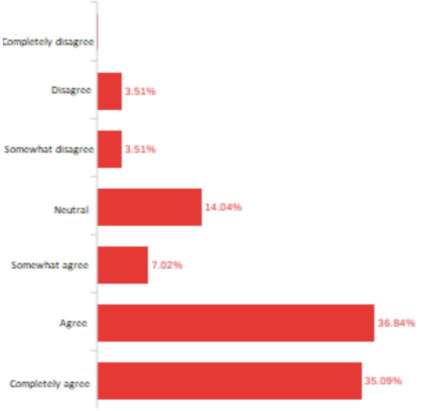

Summary of qualitative responses (n=39)

- 71.9% of the respondents agreed or completely agreed with the statement.
- Part of the respondents (n=7) stated that it is especially important that the medical stocking supplier globally assesses if the patient is self-reliant without an assistive device or by using a resistance-reducing device, and provides a single training and instruction moment. The patient needs to be referred to the occupational therapist to train more advanced assistive devices.
- Two professionals suggest that this assessment should be done by the occupational therapist.
- Some respondents (n=5; occupational therapists, general practitioners, and home care nurses) suggest that the medical stocking supplier should contact the home care nurse (if involved) to achieve further information regarding the patient's cognitive- and physical functioning before assessing the patient.

Statement 11 (minor adaptation): The medical stocking supplier needs to assess the patient's ability to maintain self-reliance without the use of an assistive device or by

using a resistance-reducing device, and provide a single training and instruction moment.

Statement 12: The medical stocking supplier needs to instruct and train the patient in using an assistive device. If it appears that the patient is not functioning self-reliant at this time, the medical stocking supplier needs to assess whether additional training is useful

Respons (n=57)

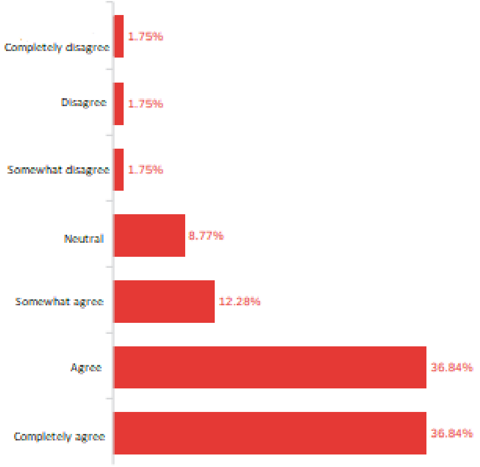

Summary of qualitative responses (n=17):

- 73.7% of the respondents agreed or completely agreed with the statement of whom 62.5% of medical stocking suppliers and 66.7% of occupational therapists.
- One medical stocking supplier indicated that with the current reimbursement system, reward, and available time, it is difficult to extensively train the patient in how to use an assistive device.
- Two respondents (one patient and one home care nurse) indicated that in current practice, some patients are referred back to the general practitioner or home care organization without any information or training in using an assistive device.

Statement 12 (minor adaptation): If it appears that the patient is not functioning self-reliant without an assistive device or with a resistance reducing assistive device, the medical stocking supplier needs to assess whether additional training is useful

<u>Follow-up*</u>

Statement 15: For patients with chronic venous disease, it is important to schedule a follow-up appointment with the treating physician after the elastic compression stocking is delivered

Respons (n=52)

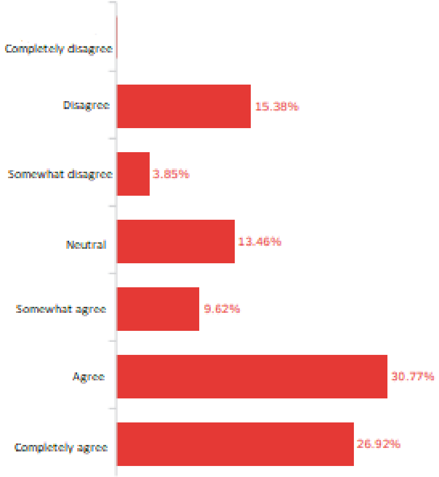

Summary of qualitative responses (n=25):

- 57.7% of the respondents agreed or completely agreed with the statement of whom 88.3% of patients.
- A large part of respondents stated that performing follow-up is important to assess treatment effects, evaluate sufficient use of the ECS, and enhance the patient's motivation and compliance therapy.
- One respondent suggested that follow-up could also be provided by the general practitioner instead of the treating physician (in case it is a dermatologist or internist), and follow-up is only necessary for the first ECS prescription.
- Another part of respondents from different disciplines (n=4 of whom one patient) indicated that follow-up is not necessary if patients can recognize problems themselves (and inform their physician) or if there are no problems.
- Some respondents from different disciplines (n=3) stated that follow-up should be performed by the medical stocking supplier.

Statement 15 (minor adaptation): For patients with chronic venous disease, it is important to schedule a follow-up appointment with the treating physician after the elastic compression stocking is delivered (only for the first prescription)

Statement 16: It is important to individualize deep venous thrombosis patients' treatment duration with elastic compression stockings based on a risk assessment using Villalta scores with a minimum treatment duration of six months

Respons (n=45)

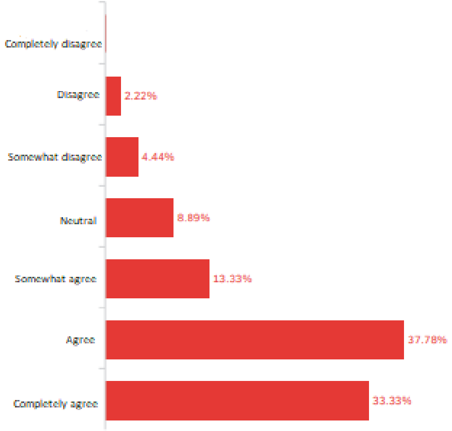

Summary of qualitative responses (n=18):

- 71.1% of the respondents agreed or completely agreed with the statement.
- Some respondents (n=3, medical stocking supplier and internists) indicated that there is sufficient evidence to individualize the treatment duration based on Villalta scores.
- One respondent (dermatologists) is concerned about the long-term effects of this strategy in developing post-thrombotic syndrome.
- Two professionals (internist and general practitioner) are not convinced that a treatment duration of six months is necessary for all patients.
- Two professionals (dermatologists) suggested adding a duplex to the follow-up in addition to assessing Villalta scores.

Statement 16 (without adaptation): It is important to individualize deep venous thrombosis patients' treatment duration with elastic compression stockings based on a risk assessment using Villalta scores with a minimum treatment duration of six months

New barrier statements round 2*

1. There is a lack of knowledge among treating physicians regarding different types of initial compression therapy
2. There is a lack of knowledge among treating physicians to inform patients about assistive devices at the moment of diagnosis
3. There is a lack of knowledge among treating physicians to determine the appropriate strength and type (circular or flat-knit) elastic compression stocking
4. Variable reimbursement criteria are a barrier to optimally select initial compression therapy and assistive devices
5. The administrative burden of achieving reimbursement for assistive devices is a barrier to optimal selection
6. Home care nurses and staff applying multilayer compression bandages at the general practice lack expertise to apply them with appropriate quality which extends the duration of initial compression therapy

Thank you for filling out our survey. We will process all answers and soon invite you for the last round of this analysis.

* All statements were provided with the following answer options and an open field text box.

Strongly disagree	Disagree	Somewhat disagree	Undecided	Somewhat agree	Agree	Strongly agree	Insufficiently informed
O	O	O	O	O	O	O	O

Round 3

General information

Dear participant,

We would like to extend our gratitude to everyone for participating in the first questionnaire of our modified Delphi study. We reached consensus on 5 out of the remaining 7 statements. These statements are provided in the supplementary file.

We kindly ask you to re-assess the statements two statements that did not reach consensus in round 2. Additionally, 1 new barrier statement was added to the current questionnaire. This questionnaire will take approximately 5 minutes of your time.

Instructions:

1. Please answer all statements, you will be allowed to add comments after each statement. We encourage you to do so, especially if you do not agree with the statement.
2. If you feel like you lack the information to assess the statement, or it is not within your expertise please choose ‘insufficiently informed’
3. The maximum response time for this survey is two weeks.

Non-consensus statements round 2

The next 2 statements did not receive consensus in questionnaire 1 (defined as < 75% of participants scoring a 6 (agree) or 7 (totally agree) on the Likert score)). Both quantitative and qualitative feedback is given per statement. We ask you to re-assess the statement after reading this feedback.

Initial compression therapy*

Statement 6: The treating physician is responsible for determining the indication, the pressure class, and the type of elastic compression stocking. This information should be included in the referral to the medical stocking supplier

Respons (n=51)

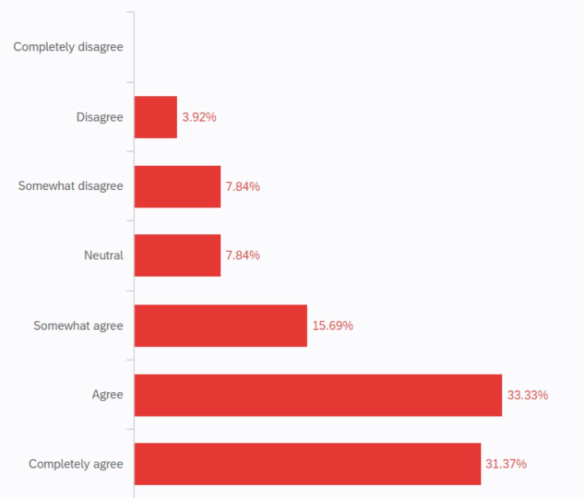

Summary of qualitative responses (n=27):

- 64.7% of the respondents agreed or completely agreed with the statement of whom 50% of the medical stocking suppliers and 71.43% of treating physicians.
- A large part of respondents (n=10) from different disciplines indicated that treating physicians (in particular general practitioners) lack the knowledge to determine the appropriate stocking type and in some cases even the appropriate pressure class. They suggested that the medical stocking supplier should determine the stocking type.
- Another part of respondents (n=4) stated that treating physicians should determine the appropriate pressure class and stocking type since they are aware of the diagnosis, contraindications, etc. Even more, since treating physicians are (as opposed to medical stocking suppliers) not exposed to financial constraints.
- Two respondents indicated that treating physicians are responsible to include this information in the referral, if they lack this knowledge, they should not treat these patient groups (n=2).
- One respondent suggested that it should be considered to prescribe adjustable compression devices instead of prescribing elastic compression stockings (by default). However, treating physicians lack knowledge regarding this type of compression.

Statement 6.1 (minor adaptation): The treating physician is responsible for determining the elastic compression stocking indication, the pressure class, and if a custom-made elastic compression stocking is necessary or if a ready-made elastic compression stocking is sufficient. This information should be included in the referral to the medical stocking supplier

In addition, there seems to be consensus that a solid referral from the treating physician to the medical stocking supplier is necessary. However, we did not yet reach consensus on the content of the referral. Therefore, we ask you to assess the next (further refined) statement independently from statement 6.1.

Statement 6.2 (major adaptation): **The treating physician is responsible for determining the elastic compression stocking indication and the pressure class, and to include this information in the referral to the medical stocking supplier**

<u>Follow-up*</u>

Statement 15: **For patients with chronic venous disease, it is important to schedule a follow-up appointment with the treating physician after the elastic compression stocking is delivered (only for the first prescription)**

Respons (n=55)

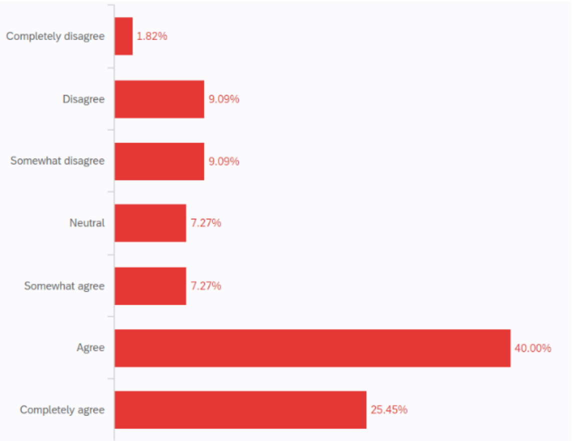

Summary of qualitative responses (n=22):

- 65.45% of the respondents agreed or completely agreed with the statement of whom 50% of the medical stocking suppliers and 57.14% of treating physicians (only 12.5% of general practitioners agreed or completely agreed).
- Five respondents from different disciplines indicated that follow-up is not necessary if patients can recognize problems themselves (and inform their physician) or if there are no problems.
- Seven respondents from different disciplines stated that follow-up appointments are necessary to evaluate the effectiveness of treatment.
- One respondent (home care nurse) indicated that a follow-up appointment should be conducted by default since it is difficult to estimate if a patient can recognize problems, which poses the risk that patients do not use the elastic compression stockings appropriately.
- Some respondents from different disciplines (n=4) indicated that follow-up appointments are not necessary by default since it is too expensive to perform follow-up only to evaluate the use of elastic compression stockings. Furthermore, treating physicians are already fully booked and experience high working pressure. This care could be provided by the medical stocking supplier or skin therapist (n=5).

Statement 15: For patients with chronic venous disease, it is important to schedule a follow-up appointment with the treating physician within several weeks after delivery of the elastic compression stocking (only for the first prescription) to check the fitting, usage and self-reliance.

New barrier statements round 3*

The need to obtain prior permission from some insurance companies necessary for the implementation of more advanced assistive devices to receive coverage, and delivery times are a barrier for optimal implementation of these devices.

Thank you for filling out our survey. We will process all answers and keep you updated regarding the progress of the protocol.

* All statements were provided with the following answer options and an open field text box.

Strongly disagree	Disagree	Somewhat disagree	Undecided	Somewhat agree	Agree	Strongly agree	Insufficiently informed
O	O	O	O	O	O	O	O

Supplementary information C: Final statements and consensus levels per questionnaire round

Statement	Round 1		Round 2		Round 3 Agreement, n (%)	
	Count	Agreement n (%)	Count	Agreement n (%)	Count	Agreement n (%)
**General statements**						
1. Active involvement of the patient and (if relevant) their informal caregiver in the decision-making process improves the probability of independence in the treatment process	59	55 (93)	NA		NA	
2. It is important to improve collaboration and dissemination of knowledge among health care professionals involved in elastic compression therapy	59	53 (90)	NA		NA	
**Initial compression therapy**						
3. Both patients with deep venous thrombosis and chronic venous disease (with edema) need to receive initial compression therapy	50	38 (76)	NA		NA	
4. The treating physician should structurally ask patients about their goals and wishes regarding self-reliance in the process and considers them in the selection of a specific type of initial compression therapy	58	45 (78)	NA		NA	
5. The treating physician needs to provide general information regarding the options of using assistive devices to maintain self-reliance during the use of elastic compression stockings at the time of diagnosis (either written or oral)	57	33 (58)	57	43 (75)	NA	
6. The treating physician is responsible for determining the indication, the pressure class, and the type of ECS. This information should be included in the referral to the medical stocking supplier	55	35 (64)	51	33 (65)	See 6.1 and 6.2	
6.1. The treating physician is responsible for determining the indication, the pressure class and to determine if custom made or standard stockings are indicated.	N.A.		N.A.		51	21 (41)
6.2. The treating physician is responsible for determining the indication and pressure class of ECS. This information should be included in the referral to the medical stocking supplier.	N.A.		N.A.		53	42 (79)
7. For patients who do not need home care assistance, the medical stocking supplier needs to assess the presence of edema during the use of initial compression therapy before fitting the ECS*	56	16 (29)	57	44 (77)	NA	
8. If home care nurses are involved to apply and remove the initial compression therapy, they are responsible to assess whether the edema has disappeared in direct consultation with the medical stocking supplier. And to subsequently instruct the patient to contact the medical stocking supplier	54	41 (76)	NA		NA	
**Onset of the elastic compression stocking and implementation of assistive devices**						
9. The medical stocking suppliers office should explicitly ask for the presence of edema during the first telephonic contact with the patient	53	40 (76)	NA		NA	
10. At the moment the elastic compression stocking is delivered, a physical follow-up appointment with the medical stocking supplier needs to take place to fit the stocking and discuss possibilities for self-reliance	57	52 (91)	NA		NA	

11. The medical stocking supplier is primarily responsible for assessing the patient's ability to maintain self-reliance in using an assistive device	57	41 (72)	56	47 (84)	NA	
12. The medical stocking supplier needs to instruct and train the patient in using an assistive device. If it appears that the patient is not functioning self-reliant at this time, the medical stocking supplier needs to assess whether additional training is useful	57	42 (74)	56	45 (80)	NA	
13. It is the medical stocking suppliers (primary) responsibility to discuss the referral to the occupational therapist for additional training with patients who are not directly functioning self-reliant after instruction and training of an assistive device	56	43 (77)	NA		NA	
14. The most suitable approach to select an assistive device is based on the estimated patient's physical characteristics and cognitive functioning; goals and wishes; and (if relevant) possibilities to involve the informal caregiver in the process	54	48 (89)	NA		NA	
**Follow-up**						
15. For patients with chronic venous disease, it is important to schedule a follow-up appointment with the treating physician within several weeks after the elastic compression stocking is delivered (only for the first prescription) to check the fitting, adherence to therapy and self-reliance. The treating physician can delegate this care to another qualified health care professional.*	52	30 (58)	55	36 (66)	56	30 (54)
16. It is important to individualize deep venous thrombosis patients' treatment duration with elastic compression stockings based on a risk assessment using Villalta scores with a minimum treatment duration of six months	45	32 (71)	43	36 (84)	NA	
17. Patients with deep venous thrombosis need to have follow-up appointments with the treating physician until the treatment duration with elastic compression stockings is established (generally after 6 or 12 months)	53	43 (81)	NA		NA	
18. If the treating physician changes from secondary to primary care during the treatment period, the treating physician should send a letter to the general practitioner which minimally includes the advised treatment duration	55	46 (84)	NA		NA	
19. All patients should have annual physical follow-up appointments with the medical stocking supplier (as long as the treatment indication lasts) to check and (if necessary) re-measure the elastic compression stocking	55	46 (84)	NA		NA	
20. If the treating physician changes from secondary to primary care during the treatment period, the first treating physician should inform the patient, general practitioner, and home care organization (if involved)	57	43 (75)	NA		NA	
21. When elastic compression therapy needs to be discontinued, the treating physician should inform the patient, the medical stocking supplier, and the home care organization (if involved)	57	46 (81)	NA		NA	

* These statements underwent major modifications throughout the questionnaire rounds as presented in Supplementary information B.
